# Excessive Ultrafiltration Associates with EPO Hyporesponsiveness in Elderly Chronic Hemodialysis Patients

**DOI:** 10.3390/biomedicines14030497

**Published:** 2026-02-25

**Authors:** Luís Belo, Maria João Valente, Susana Rocha, Susana Coimbra, Cristina Catarino, Elsa Bronze-da-Rocha, Petronila Rocha-Pereira, Maria do Sameiro-Faria, José Gerardo Oliveira, João Carlos Fernandes, Vasco Miranda, Alice Santos-Silva

**Affiliations:** 1 UCIBIO i4HB, Faculdade de Farmácia, Universidade do Porto, Rua Jorge Viterbo Ferreira 228, 4050-313 Porto, Portugal; mjoao.pcv@gmail.com (M.J.V.); srocha@ff.up.pt (S.R.); cristinacatarino@ff.up.pt (C.C.); elsa.rocha@ff.up.pt (E.B.-d.-R.); petronila@live.com.pt (P.R.-P.); mariasameirofaria@gmail.com (M.d.S.-F.); assilva@ff.up.pt (A.S.-S.); 2 UCIBIO i4HB, Translational Toxicology Research Laboratory, University Institute of Health Sciences (1H-TOXRUN, IUCS-CESPU), Avenida Central de Gandra 1317, 4585-116 Gandra, Portugal; 3 Hemodialysis Clinic of Felgueiras, CHF, 4610-106 Felgueiras, Portugal; 4 Hemodialysis Clinic of Porto, CHP, 4200-277 Porto, Portugal; gccot@hsjoao.min-saude.pt; 5 Center for Health Technology and Services Research (CINTESIS), Faculty of Medicine, University of Porto, 4200-319 Porto, Portugal; 6 NefroServe Hemodialysis Clinic of Viana do Castelo, 4901-858 Viana do Castelo, Portugal; joaoqarlos@gmail.com; 7 Hemodialysis Clinic of Gondomar, CHD, 4420-086 Gondomar, Portugal; mail@vascomiranda.com

**Keywords:** end-stage kidney disease, hemodialysis, elderly, anemia, ultrafiltration

## Abstract

**Background**: The population of elderly patients undergoing chronic hemodialysis is increasing, and anemia represents a frequent complication. The aim of our study was to evaluate the association between ultrafiltration rate (UFR) in hemodialysis and erythropoietin (EPO) response in elderly patients with end-stage kidney disease (ESKD). **Methods**: This was a multicenter, retrospective observational study, involving elderly patients (aged 65 years or more) under chronic hemodialysis therapy. Individuals were divided into two groups according to the UFR adjusted to weight (UFR/W): lower (UFR-N) or higher (UFR-H) than 10 mL/h/kg. EPO resistance index (ERI) was calculated. We evaluated the hemogram, reticulocyte count, and quantified markers of iron metabolism and inflammation. **Results**: A total of 193 patients were enrolled in the study: 141 patients met criteria for inclusion in UFR-N group and 52 in UFR-H group. Compared to UFR-N, patients in the UFR-H group presented significantly higher doses of erythropoiesis-stimulating agents (ESA) and ERI values, with similar hemoglobin (Hb) and inflammatory markers levels. In a sub-analysis, within patients presenting transferrin saturation (TSAT) lower than 20%, a more marked difference in ERI between UFR groups was observed, being much higher in UFR-H compared with UFR-N. In this subgroup (UFR-H with lower TSAT), levels of hepcidin were lower than in the other subgroups. **Conclusions**: Our data show that UFR appears to be a contributing factor of ESA response in elderly patients under hemodialysis, particularly in those with lower iron availability. These findings suggest that inadequate weight control and/or UF prescription seem to aggravate ESA needs to achieve target Hb.

## 1. Introduction

Anemia remains one of the most prevalent and clinically significant complications in patients undergoing chronic hemodialysis, contributing to reduced quality of life, increased cardiovascular morbidity, and higher mortality [1,2]. Despite advances in erythropoiesis-stimulating agents (ESA) and iron supplementation strategies, a substantial proportion of hemodialysis patients exhibit hyporesponsiveness to anemia management [3]. This phenomenon reflects the multi-factorial nature of impaired erythropoiesis in end-stage kidney disease (ESKD), involving inflammation, iron dysregulation, and alterations in bone marrow function.

The elderly population is a highly representative subgroup in hemodialysis patients [4]. In this particularly vulnerable subgroup, the interaction between ultrafiltration rate (UFR) and erythropoiesis may be amplified. Aging is accompanied by “inflammaging,” a state of chronic, low-grade systemic inflammation driven by immunosenescence, oxidative stress, and persistent antigenic stimulation [5]. Concurrently, bone marrow senescence leads to reduced hematopoietic stem cell reserve, impaired erythroid differentiation, and increased susceptibility to inflammatory inhibition of erythropoiesis [6]. In this context, dialysis-related stressors such as aggressive ultrafiltration may further compromise marrow function and contribute to ESA resistance.

UFR is a central yet underappreciated determinant of hemodialysis tolerance and systemic homeostasis. Excessive UFR has been consistently associated with intradialytic hypotension, myocardial stunning, gut ischemia, and increased mortality [7]. Beyond hemodynamic instability, high UFR may promote tissue hypoxia, endothelial dysfunction, and activation of pro-inflammatory pathways, thereby exacerbating the chronic inflammatory milieu characteristic of ESKD. Such effects may have direct and indirect consequences on erythropoiesis, influencing both endogenous red blood cell production and responsiveness to ESAs. It was demonstrated that a higher UFR associates with greater resistance to erythropoietin (EPO) treatment, but the influence of age was not addressed [8].

This study aims to investigate the association between UFR and erythropoietic response in chronic hemodialysis patients, with a specific focus on elderly individuals. This may help to optimize anemia management and improve outcomes in this growing patient population.

## 2. Materials and Methods

### 2.1. Patients

All procedures were conducted in accordance with the principles of the Declaration of Helsinki (1964), as revised in 2008. The study protocol and data analysis were approved by the National Data Protection Commission (Proc. No. 762/2017; Authorization No. 532/2017) and by the Ethics Committee of the Faculty of Pharmacy, University of Porto (Report No. 26-04-2016). Written informed consent was obtained from all participants prior to inclusion in the study.

**Figure 1:**
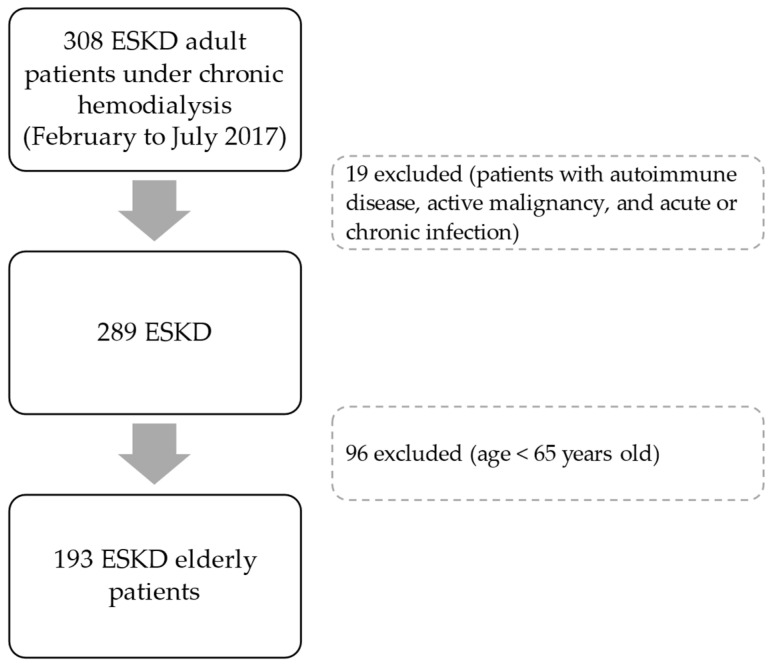
Flow-chart of the end-stage kidney disease (ESKD) patient selection.

This is a retrospective study of a previous work involving adult patients under dialysis therapy for at least 90 days, selected during the period from February to July 2017, from 5 dialysis clinics in the Northern region of Portugal [4]. Patients were clinically evaluated by nephrologists, and blood was collected before the midweek dialysis session for analytical studies. Data regarding demographic characteristics, medical history, dialysis, and pharmacological prescriptions were collected. Patients with known active malignancy, autoimmune diseases, and acute or chronic infection were excluded (Figure 1). In this retrospective study, only elderly patients—meeting the criteria of an age equal to or higher than 65 years—were included (*n* = 193; Figure 1). The elderly definition that we used is in accordance with PORDATA—The Database of Contemporary Portugal.

The ultrafiltration (UF) value for the dialysis session on the day of blood collection was obtained for each participant. UFR was then calculated using treatment length (usually 4 h) and adjusted to patient weight (UFR/W). Patients were divided into two groups according to the UFR/W value: <10 mL/h/kg (UFR-N) and ≥10 mL/h/kg (UFR-H). We applied the UFR/W cut-off of 10 mL/kg/h, as this a safety threshold commonly recommended in good clinical practice to reduce the risk of hemodynamic instability and mortality.

### 2.2. Erythropoiesis-Stimulating Agents (ESA) and Iron Therapies

Treatment with intravenous iron and recombinant human erythropoietin (rhEPO) was in accordance with the European Renal Best Practice Guidelines [9].

ESA prescription included the following drugs: darbepoetin α (Aranesp^®^; μg), epoetin α (Eprex^®^; IU), and epoetin β (Neorecormon^®^; IU). The doses of darbepoetin α were converted to standardized equivalent doses of epoetin, according to the World Health Organization (WHO) daily defined dose (DDD): 4.5 μg of darbepoetin α are equivalent to 1000 IU of epoetin (conversion factor: 1:222) [10]. EPO resistance index (ERI) was calculated by the formula: EPO dose per week (IU)/body weight (kg)/hemoglobin (g/dL). Patients on iron therapy used iron sucrose (Venofer^®^).

### 2.3. Assays

Blood samples were obtained immediately prior to the dialysis procedure and processed within 2 h of collection. Specimens were collected into tubes containing K3-EDTA as anticoagulant and into anticoagulant-free tubes to allow the preparation of whole blood and serum. Serum aliquots were promptly separated and stored at −80 °C until analysis.

Erythrocyte, hemoglobin concentration, and hematocrit values were evaluated by using an automatic blood cell counter (Sysmex K1000; Sysmex, Hamburg, Germany). Reticulocytes were quantified by microscopic counting on blood smears, after vital staining with new methylene blue (Reticulocyte stain; Sigma-Aldrich Co. LLC., St. Louis, MO, USA).

Serum iron level was evaluated using a colorimetric method (Iron, Randox Laboratories Ltd., North Ireland, UK). Serum ferritin and serum transferrin were measured by immunoturbidimetry (Ferritin and Transferrin, Randox Laboratories Ltd., North Ireland, UK). Transferrin saturation (TSAT) was calculated by the formula: TSAT (%) = 70.9 × serum iron concentration (µg/dL)/serum transferrin concentration (mg/dL). Levels of hepcidin were evaluated by using a commercially available enzyme-linked immunosorbent assay (ELISA) kit (Human Hepcidin Quantikine ELISA Kit, R&D Systems, Minneapolis, MI, USA).

Two cytokines were measured in serum: interleukin (IL)-6 and tumor necrosis factor-alpha (TNF-α) by ELISA kits (Human IL-6 Quantikine HS and Human TNF-alpha Quantikine HS, R&D Systems).

Creatinine, urea, phosphorus and albumin were quantified using standardized automated routine assays (Roche Diagnostics, Basel, Switzerland).

To assess dialysis adequacy, an additional blood sample was collected after the hemodialysis session to calculate the urea reduction ratio (URR) and the urea clearance index Kt/V, where *K* represents urea clearance, *t* the duration of the dialysis session, and *V* the patient’s urea distribution volume. The URR was calculated using the formula (1 − [Ct/Co]) × 100, where *Ct* and *Co* denote post-dialysis and pre-dialysis serum urea concentrations, respectively. The Daugirdas’s formula was used to calculate eKt/V [11].

### 2.4. Statistical Analysis

The normality of data distribution was assessed using the Kolmogorov–Smirnov test. Variables with a normal distribution are reported as mean ± standard deviation (SD), whereas non-normally distributed variables are expressed as median [interquartile range]. Group comparisons were performed using the chi-squared test or Fisher’s exact test for categorical variables, and the unpaired Student’s *t* test or the Mann–Whitney *U* test for continuous variables, as appropriate. Adjustment for confounding factors (e.g., body mass index (BMI), sex, age) was performed using analysis of covariance, following log transformation of variables when appropriate. The strength of the association between the variables studied was assessed using Spearman’s rank correlation coefficient. To assess the independent relationship of various factors with ERI, multiple regression analysis was conducted (following the log transformation of the non-normally distributed variables, through stepwise selection, applying an entry threshold of *p* < 0.05). Statistical analyses were conducted using the IBM Statistical Package for the Social Sciences (SPSS) for Windows (version 30.0; IBM Corp., Chicago, IL, USA). A *p* value < 0.05 was considered statistically significant.

## 3. Results

### 3.1. Demographic and Clinical Data of Patients

We analyzed 193 elderly ESKD patients under chronic dialysis therapy with a mean age of 76.7 ± 7.0 years; 141 patients (73%) were classified as having appropriate UFR (UFR-N) and 52 (27%) as UFR-H. UFR groups did not differ in terms of age. UFR-H had a higher prevalence of male patients and presented lower BMI values (Table 1). Arterial hypertension was present in 116 individuals (60.1%), although the number of cases did not differ statistically between groups (58.2% for UFR-N and 65.4% for UFR-H, *p* = 0.410).

Patients were under dialysis therapy three times per week, for a median period of 4 h, and used high-flux polysulfone FX-class dialyzers (1.4–2.2 m^2^) of Fresenius (Bad Homburg, Germany). Arteriovenous fistula was the most common vascular access used (*n* = 161, 83.4%), with a similar number of patients in both groups (84.4% for UFR-N and 80.8% for UFR-H), while online hemodiafiltration was the most common dialysis modality (*n* = 162, 83.9%) with slightly higher prevalence of this modality in UFR-N (87.2%) than in UFR-H (75.0%). All remaining patients were under high-flux hemodialysis. During dialysis treatments, patients were given unfractionated heparin (UFH) as an anticoagulant, according to weight, previous dialysis events (coagulation and presence of fibers on dialyzers) and chronic anticoagulation medication.

The main causes of renal failure were diabetes mellitus (*n* = 75) and other/uncertain etiology (*n* = 75). UFR groups were equilibrated for chronic kidney disease (CKD) etiology.

The majority of patients were under ESA (83.9%), and intravenous iron (62.2%) therapies. The relative number of patients under these pharmacological therapies was similar between groups. Iron doses were also similar between the two groups, but ESA doses and ERI values were substantially higher in UFR-H group (Table 1). The differences for ERI maintained statistical significance after adjustment for BMI, age and sex (*p* = 0.001). Nevertheless, in both subgroups, females exhibited significantly higher ERI values than males: UFR-N (7.33 [4.12–13.60] vs 5.03 [2.76–8.66], *p* = 0.001) and UFR-H (14.91 [10.54–18.23] vs 7.71 [3.48–16.00], *p* = 0.028).

Serum levels of phosphorus, creatinine, albumin, and urea were similar between groups (Table 1).

### 3.2. Hematological and Biochemical Data of Patients

No differences were found for hematological and biochemical parameters between UFR groups, except for transferrin, that was lower in UFR-N group (Table 2).

Among patients with ESA therapy (*n* = 162), ERI values were negatively correlated with albumin (r = −0.162, *p* = 0.039), serum iron (r = −0.433, *p* < 0.001) and TSAT (r = −0.420, *p* < 0.001) levels, and positively correlated with IL-6 concentration (r = 0.249, *p* = 0.001) and UFR/W (r = 0.168, *p* = 0.033). In multiple linear regression analysis, sex, TSAT, IL-6 levels, and UFR/W values remained statistically associated with ERI values (Table 3).

**Figure 2:**
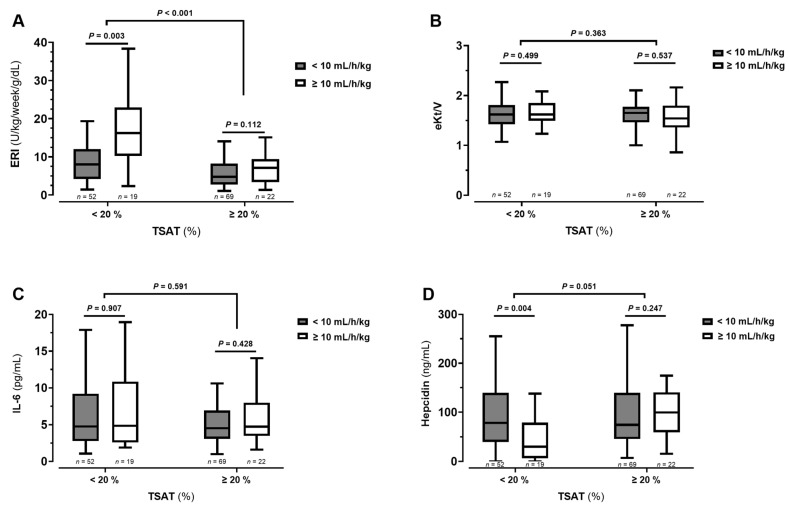
Values of erythropoietin resistance index (ERI; panel **A**), eKt/V (panel **B**), interleukin-6 (IL-6; panel **C**) and hepcidin (panel **D**) levels in elderly hemodialysis patients taking ESAs (*n* = 162), according to transferrin saturation (TSAT) and ultrafiltration rate adjusted to weight (UFR/W) cut-off values. The interquartile range is represented by the boxes, with the upper and lower edges denoting the 75th and 25th percentiles, respectively. The median levels are shown by the central horizontal lines inside the boxes. Differences between groups were evaluated by the Mann–Whitney U test. A *p* < 0.05 was considered as statistically significant.

We further analyzed results according to iron status. For each UFR group, we further divided patients into two subgroups according to TSAT values: lower than 20% or equal/higher than 20%. Globally, ERI values were higher in patients with low TSAT than in those with adequate TSAT (*p* < 0.001; Figure 2A). Within the low TSAT subgroup, ERI values were higher (*p* = 0.003; Figure 2A) and hepcidin were lower (*p* = 0.006; Figure 2D) in those with high UFR (UFR-H). No significant differences between TSAT groups were found for eKt/V (Figure 2B) and IL-6 (Figure 2C).

## 4. Discussion

The relationship between UFR and anemia outcomes has not been adequately explored in ESKD patients under hemodialysis therapy. In the present work, we report evidence suggesting that dialysis prescription parameters, including UFR, may influence ESA response. We found in elderly patients under chronic hemodialysis that excessive UFR is associated with higher ERI, particularly in non-iron repleted cases.

In our study, patient groups presented similar age, dialysis vintage and CKD etiology. Both groups presented adequate hemoglobin levels, but the UFR-H group needed higher ESA doses to achieve target values, and therefore, ERI values were substantially higher in these patients (Figure 2). The effect was more pronounced in females than in males, which is consistent with previous evidence indicating that female sex is associated with greater EPO resistance, likely mediated by hormonal differences as well as a higher prevalence of iron metabolism dysfunction [12]. Ou results obtained by multiple regression showed that sex and UFR/W are independently associated with ERI. Modeling UFR/W as a continuous variable revealed that increasing UFR/W values were associated with progressively greater hyporesponsiveness to EPO.

Elderly CKD patients often present loss of appetite and better ingestion control, resulting in lower UF values in dialysis sessions compared with younger patients [4]. A high UFR may be particularly demanding in elderly population—due to age-related cardiovascular modifications—leading more frequently to episodes of hypotension and consequently lower organ perfusion [7]. Pathologic hypoxia of bone marrow promotes a pro-inflammatory phenotype, activating leukocytes [13] and inhibiting erythropoiesis. In elderly individuals an increased susceptibility to inflammation-induced inhibition of erythropoiesis may occur, due to bone marrow senescence. Inflammation can contribute to ESA response, and in the present study IL-6 levels correlated positively with ERI. However, we were unable to find differences in circulating values of IL-6 (or TNF-α) between UFR groups. IL-6 therefore does not appear to be associated with the effect of UFR. Nevertheless, we cannot exclude the potential involvement of other inflammatory mediators (namely those involved in other signalization vias), a local exacerbated inflammation process without systemic expression, or even non-inflammatory mechanisms. In addition, it cannot be ruled out that residual confusion may be a possible reason for a missing association between markers of inflammation and UFR, since both are complex, multi-factorial, and deeply influenced by several factors/conditions

Malnutrition could also influence the results that we obtained for ERI, as it may limit response to ESA [14]. Patients with anorexia and cachexia are particularly vulnerable and usually present nutrient deficiencies [15]. Despite lower BMI values in the UFR-H group, the levels of albumin, urea, phosphorus and creatinine were similar between the two groups. Furthermore, all patients were supplemented with folic acid and other B-complex vitamins, after dialysis sessions. Thus, we believe that this was not a strong contributor factor for the differences obtained with ERI between groups.

Finally, as iron availability is a major driven factor of ESA response (corroborated by our own results), we performed sub-analyses in patients with adequate TSAT values (≥20%) and in those not reaching target TSAT. As expected, ERI values were higher in patients with lower TSAT (Figure 2A). However, the association of higher UFR with EPO hyporesponsiveness was observed in both TSAT subgroups, even though the difference was only statistically significant in those not achieving TSAT target (<20%). Thus, ERI values seem to be independently determined by both iron availability and UFR values.

Hepcidin is a masterpiece in regulating iron metabolism and inflammation is known to stimulate hepcidin synthesis [16]. In cases of inflammation, increased hepcidin synthesis inhibits iron absorption and availability for erythropoiesis. In the present study, hepcidin levels were similar between UFR groups (Table 1), but were lower in the subgroup of UFR-H patients with lower TSAT (Figure 2D). UFR subgroups were matched for IL-6 levels (the major trigger for hepatic hepcidin synthesis) and, therefore, inflammation does not explain hepcidin differences (Figure 2C). It was also not apparently related to greater dialysis removal in UFR-H group. A previous study reported that hemodialysis reduces circulating hepcidin, being positively correlated with dialysis dose (spKt/V) [17], but our groups were matched for eKt/V (Figure 2B). The lower hepcidin levels that we observed in UFR-H subgroup are likely to be related to the higher ESA doses received by these patients. It is known that the erythropoietic stimulus with ESA decreases hepcidin expression [18]. The presumed tissue hypoxia in patients with high UFR and with low TSAT, may justify the particularly higher ESA doses which consequently lower hepcidin to try to compensate (in)disponible iron for erythropoiesis. However, it should not be overlooked that hepcidin levels are highly dynamic, regulated by a complex interplay of factors, and we did not perform excretion studies.

This study has some limitations that should be acknowledged. First, its retrospective and cross-sectional design precludes causal inference and limits the assessment of temporal and dose–response relationships between the studied variables. Second, the relatively small number of patients in the UFR- and TSAT-defined groups reduces statistical power and may have limited the ability to detect clinically relevant differences. Nevertheless, we believe that our regression analysis using the TSAT and UFR as continuous variables may have overcome this limitation. Additionally, we evaluated only a restricted panel of circulating inflammatory markers, which may not fully capture the complexity of inflammation, particularly at the local bone marrow level. Finally, we did not quantify specific nutrients involved in erythropoiesis, such as key vitamins or micronutrients, which may represent unmeasured confounders influencing hematologic outcomes.

## 5. Conclusions

This study provides evidence that inadequate weight control and UFR prescription may be associated with EPO hyporesponsiveness, in elderly patients under chronic dialysis. This inappropriate response appears to be particularly notorious in patients not achieving target TSAT values in hemodialysis setting. High UFR is likely to be a stressor for bone marrow, limiting erythropoiesis, but the independent contribution of UFR to the observed EPO hyporesponsiveness deserves further clarification.

## Figures and Tables

**Table 1:** Demographic, clinical, and dialysis-related data in total patients and UFR groups in elderly end-stage kidney disease patients under chronic dialysis.

	Total	UFR-N (<10 mL/h/kg)	UFR-H (≥10 mL/h/kg)	*p*
Number	193	141	52	
Age, years	76.7 ± 7.0	77.2 ± 7.0	75.3 ± 6.9	0.111
Sex male, *n* (%)	103 (53.4)	68 (48.2)	35 (67.3)	0.023
BMI, Kg/m^2^	25.9 ± 4.4	26.4 ± 4.5	24.8 ± 4.1	0.023
Systolic blood pressure (mm Hg)	137.5 ± 19.5	136.8 ± 19.6	139.5 ± 19.2	0.384
Diastolic blood pressure (mm Hg)	58.8 ± 10.9	58.2 ± 10.9	60.6 ± 10.6	0.180
Etiology of CKD, *n* (%)				
Diabetic Nephropathy	75 (38.9)	53 (37.6)	22 (42.3)	0.740 *
Hypertensive Nephrosclerosis	25 (13.0)	17 (12.1)	8 (15.4)	
Polycystic Kidney Disease	10 (5.2)	9 (6.4)	1 (1.9)	
Chronic Glomerulonephritis	8 (4.1)	5 (3.5)	3 (5.8)	
Other or Undetermined	75 (38.8)	57 (40.4)	18 (34.6)	
Dialysis Vintage, Years	3.79 [1.61–7.51]	3.79 [1.54–6.75]	3.92 [1.72–8.25]	0.579
Intradialytic therapy				
ESA Prescription, *n* (%)	162 (83.9)	121 (85.8)	41 (78.8)	0.271
ESA Dose (IU/Kg/week)	77.7 [42.2–135.4]	64.4 [40.4–114.3]	115.4 [65.5–188.7]	0.002
ERI (IU/Kg/week/g/dL)	6.84 [3.72–11.50]	5.85 [3.45–9.96]	9.59 [5.37–18.15]	0.003
Iron Prescription, *n* (%)	120 (62.2)	89 (63.1)	31 (59.6)	0.738
Iron dose (mg/week)	35.0 [25.0–57.5]	30.0 [25.0–50.0]	50.0 [30.0–100.0]	0.089
Biochemical and Dialysis Markers				
Phosphorus, mg/dL	3.90 [3.30–4.61]	3.87 [3.28–4.60]	3.91 [3.41–4.87]	0.340
Creatinine, mg/dL	7.55 ± 2.00	7.45 ± 2.10	7.83 ± 1.82	0.076
Albumin, g/dL	3.8 [3.6–4.0]	3.8 [3.5–4.1]	3.8 [3.6–4.0]	0.706
Urea, mg/dL	111 [92–141]	110 [90–141]	122 [96–142]	0.181
URR, %	80.0 [76.0–83.0]	80.0 [76.5–83.0]	79.0 [75.0–83.0]	0.488
eKt/V	1.62 ± 0.28	1.62 ± 0.28	1.62 ± 0.30	0.918
UF, L	2.2 [1.6–2.8]	1.9 [1.5–2.4]	3.0 [2.6–3.5]	<0.001
UFR/W (mL/h/kg)	8.0 [5.9–10.2]	7.0 [5.4–8.3]	11.2 [10.7–12.9]	<0.001

Data presented as mean ± standard deviation or as median [interquartile range], unless otherwise indicated. *p* value for comparisons between UFR-N and UFR-H groups; * comparison of frequency distribution of variables (χ^2^); BMI—body mass index; CKD—chronic kidney disease; ERI—erythropoietin resistance index; ESA—erythropoiesis-stimulating agents; UF—ultrafiltration; UFR/W—ultrafiltration rate adjusted to weight; URR—urea reduction ratio.

**Table 2:** Hematological and biochemical data in total patients and UFR groups in elderly end-stage kidney disease patients under chronic hemodialysis.

	Total	UFR-N (<10 mL/h/kg)	UFR-H (≥10 mL/h/kg)	*p*
Hematological Data				
Erythrocytes (×10^12^/L)	3.71 [3.43–4.01]	3.71 [3.43–3.98]	3.70 [3.40–4.06]	0.997
Hemoglobin (g/dL)	11.4 [10.6–12.2]	11.3 [10.6–12.2]	11.4 [10.5–11.9]	0.983
Hematocrit (%)	35.1 [32.8–37.4]	35.3 [32.6–37.5]	34.9 [32.8–37.1]	0.662
Reticulocytes (%)	1.1 [0.6–1.5]	1.1 [0.7–1.5]	1.1 [0.6–1.6]	0.926
Reticulocytes (×10^9^/L)	38.9 [25.4–57.4]	39.7 [26.2–54.9]	37.6 [23.0–57.9]	0.783
Iron metabolism markers				
Iron (µg/dL)	54.0 [45.0–74.0]	54.0 [45.0–73.5]	60.0 [45.3–74.7]	0.550
Transferrin (mg/dL)	180 [164–209]	178 [162–200]	195 [176–218]	0.003
TSAT (%)	22.0 [16.7–27.6]	21.8 [16.8–27.9]	22.6 [16.0–27.6]	0.794
Ferritin (ng/mL)	342 [218–503]	344 [229–521]	314 [192–482]	0.180
Hepcidin (ng/mL)	77.5 [43.3–138.0]	77.0 [44.1–139.4]	79.0 [39.7–135.8]	0.484
Inflammatory markers				
IL-6 (pg/mL)	4.63 [3.00–7.62]	4.56 [2.97–7.62]	4.80 [3.30–7.68]	0.521
TNF-α (pg/mL)	3.49 [2.70–4.70]	3.57 [2.74–5.06]	3.38 [2.55–4.54]	0.250

Data presented as median [interquartile range]. *p* value for comparisons between UFR groups. IL-6, interleukin-6; TNF-α, tumor necrosis factor-alpha; TSAT, transferrin saturation; UFR, ultrafiltration rate.

**Table 3:** Main variables associated with erythropoietin resistance index (ERI) in elderly chronic dialysis patients by multiple linear regression analysis.

Dependent Variable	Model	Unstandardized Coefficients		Standardized Coefficients	t	*p*
		B	Std. Error	Beta		
Ln ERI	(Constant)	3.459	0.545		6.342	<0.001
	Ln TSAT	−0.840	0.143	−0.399	−5.859	<0.001
	Ln IL-6	0.231	0.078	0.196	2.944	0.004
	Sex	−0.349	0.116	−0.204	−2.996	0.003
	Ln UFR/W	0.402	0.143	0.187	2.804	0.006

R^2^ for multivariable regression model = 0.293. Sex was introduced as a dichotomic variable (females = 0, males = 1). IL-6, interleukin-6; TSAT, transferrin saturation; UFR/W—ultrafiltration rate adjusted to weight.

## Data Availability

The original contributions presented in this study are included in the article. Further inquiries can be directed to the corresponding authors.
